# Improving anti-tumor activity of sorafenib tosylate by lipid- and polymer-coated nanomatrix

**DOI:** 10.1080/10717544.2016.1245371

**Published:** 2017-02-06

**Authors:** Yang Guo, Ting Zhong, Xiao-Chuan Duan, Shuang Zhang, Xin Yao, Yi-Fan Yin, Dan Huang, Wei Ren, Qiang Zhang, Xuan Zhang

**Affiliations:** 1 Beijing Key Laboratory of Molecular Pharmaceutics and New Drug Delivery Systems, School of Pharmaceutical Sciences, Peking University, Beijing, China,; 2 Department of Pharmaceutics, School of Pharmaceutical Sciences, Peking University, Beijing, China, and; 3 State Key Laboratory of Natural and Biomimetic Drugs, Peking University, Beijing, China

**Keywords:** Sorafenib tosylate, nanomatrix, poorly water-soluble drugs, bioavailability, anti-tumor activity

## Abstract

In the present study, we select the Sylysia 350 (Sylysia) as mesoporous material, distearoylphosphatidylethanolamine-poly(ethylene glycol)_2000_ (DSPE-PEG) as absorption enhancer and hydroxy propyl methyl cellulose (HPMC) as crystallization inhibitor to prepare sorafenib tosylate (SFN) nanomitrix (MSNM@SFN) for improving the anti-tumor activity of SFN. The MSNM@SFN was prepared by solvent evaporation method. The solubility, dissolution, and bioavailability of SFN in MSNM@SFN were also investigated. The anti-tumor activity of MSNM@SFN was evaluated *in vitro* and *in vivo*. Our results indicated that the solubility and dissolution of SFN in MSNM@SFN were significantly increased. The oral bioavailability of SFN in MSNM@SFN was greatly improved 7.7-fold compared with that in SFN suspension. The enhanced anti-tumor activity of MSNM@SFN was confirmed *in vitro* and *in vivo* experiments. This nanomatrix developed in this study could be a promising drug delivery platform for improving the therapeutic efficacy of poorly water-soluble drugs.

## Introduction

Many strategies have been used to improve the solubility of poorly water-soluble drugs, including solid dispersions, soluble cyclodextrin complexes, self-emulsifying drug delivery systems, nanocrystals, ordered mesoporous silica, etc. (Singh et al., 2011). The use of mesoporous silica is one of the more rapidly developing formulation techniques for enhancing the solubility of poorly water-soluble drugs (Vialpando et al., 2011; Mamaeva et al., 2013; Santos et al., 2013; Shen et al., 2013; Xu et al., 2013; Wang et al., 2015). Their large surface area and pore volume make mesoporous silica materials excellent candidates for efficient drug loading and rapid release. The contribution for enhancing the solubility and dissolution rate of the poorly water-soluble drugs is since the small nanopores of mesoporous silica can convert a crystalline drug to an amorphous form (Ukmar et al., 2011; Wani et al., 2012). Sylysia 350 (Sylysia) is a desired mesoporous silica material with a particle size of 3.9 μm and a large number of internal pores about 21 nm which could be used as carrier material to improve the solubility of poorly water-soluble drugs (Jia et al., 2011; Wang et al., 2011; Dai et al., 2015).

Sorafenib tosylate (SFN) is an oral multi-kinase inhibitor. It was initially identified as a Raf-1 kinase inhibitor. Further *in vitro* and *in vivo* studies demonstrated that SFN inhibits cell surface tyrosine kinase receptors (e.g. VEGFR-1, VEGFR-2, VEGFR-3; PDGFR-b, c-KIT, FLT-3 and RET) and downstream intracellular serine/threonine kinases of the RAF/MEK/ERK pathway (e.g. Raf-1, wild-type B-Raf, and mutant B-Raf) leading to a dual mechanism of action by targeting tumor cell proliferation and tumor angiogenesis (Wilhelm et al., 2004; Wilhelm et al., 2006; Gadaleta-Caldarola et al., 2015).

The relative bioavailability of current marketed tablet formulation of SFN, compared to an oral solution, was 38–49% (van Erp et al., 2009). The absolute bioavailability of SFN suspension in rats was only about 8.43% (Zhang et al., 2014). SFN is very lipophilic (LogP = 3.8) and has a strong crystal lattice. The aqueous solubility of SFN in the gastrointestinal tract (GI) fluids is very low. Considering to its high permeability through the gastrointestinal membrane, SFN is classified as a BCS II compound. Therefore, strategies to improve the solubility and bioavailability of SFN are of substantial therapeutic benefit.

A nanomatrix was prepared from a mesoporous material Sylysia and a pH-sensitive polymer Eudragit to improve the solubility and bioavailability of SFN (Wang et al., 2011). Also, a nanodiamond system loading SFN-coated DSPE-PEG as an absorption enhancer to improve the bioavailability and efficacy on suppression of cancer metastasis has been reported (Zhang et al., 2014). In addition, the poly(vinylpyrrolidone-vinyl acetate) and sodium lauryl sulfate (PVP-VA/SLS) complexes have been used to increase the solubility of SFN at a lower critical aggregation concentration. The enhanced solubility provided a faster initial SFN dissolution rate, analogous to a forceful “spring” to release SFN into solution. The SLS appears to impair the ability of PVP-VA to act as an efficient “parachute” to keep the SFN in solution and maintain its supersaturation (Liu et al., 2016).

To summarize the reported references, we suggested that a nanomatrix with the combination of mesoporous material, absorption enhancer, and crystallization inhibitor would be in favor of improving the bioavailability and efficacy of SFN. Therefore, in the present study, we select the Sylysia 350 as mesoporous material, DSPE-PEG as absorption enhancer and HPMC as crystallization inhibitor to prepare SFN nanomitrix (MSNM@SFN). The characteristics and the anti-tumor activity of MSNM@SFN were evaluated *in vitro* and *in vivo*.

## Materials and methods

### Materials

SFN was purchased from MERYER Chemical Technology Co. Ltd. (Shanghai, China). Mesoporous silica (Sylysia 350) was obtained from Fuji Silysia Chemicals (Kasugai, Japan). Hydroxy propyl methyl cellulose (HPMC, 7500–14 000 mPa.s) was obtained from Alfa Aesar Chemical Co. Ltd. (Tianjin, China). Distearoylphosphatidylethanolamine-poly (ethylene glycol)_2000_ (DSPE-PEG) was provided by NOF Co. ORPORATION (Tokyo, Japan). Hoechst 332589 (blue) and FITC goat anti-rabbit secondary antibodies (1:1000) were supplied by Molecular Probes Inc. (Eugene, OR). Rabbit polyclonal CD31 antibody (10 μg/ml) was obtained from Abcam Inc. (Cambridge, MA). Leibovitz’s L-15 cell culture media, penicillin–streptomycin, fetal bovine serum (FBS), and L-glutamine were obtained from GIBCO, Invitrogen Corp. (Carlsbad, CA). All other chemicals were of analytical or HPLC grade.

### Cell lines

The MDA-MB-231 cell lines (a human breast cancer cell) were obtained from the Chinese Academy of Sciences Cells Bank (Shanghai, China) and cultivated in Leibovitz’s L-15 medium which was supplemented with 10% FBS (GIBCO, Invitrogen Corp.), 100 units/ml penicillin, and 100 μg/ml streptomycin. The cultures were maintained at 37 °C, 95% relative humidity.

### Animals

Male Sprague-Dawley (SD) rats (190–210 g) and female BALB/C nude mice (18–20 g) were obtained from the Experimental Animal Center of Peking University Health Science Center. All care and handling of the animals were performed with the approval of the Institutional Authority for Laboratory Animal Care of Peking University.

### Preparation of SFN mesoporous silica nanomatrix (MSNM@SFN)

#### Preparation of MSNM@SFN

The MSNM@SFN was prepared by solvent evaporation method. Briefly, a volume of 1 ml SFN methanol solution (10 mg/ml) was dropped into Sylysia dichloromethane suspension (3 ml, 10 mg/ml) in a round flask by sonication for 30 min, and then evaporated into dryness under reduced pressure at 40 °C. After that, a volume of 10 ml HPMC dichloromethane solution (3 mg/ml) was dropped into the mixtures and stirred for 24 h and then evaporated into dryness under reduced pressure at 40 °C. Subsequently, a volume of 3 ml of DSPE-PEG dichloromethane solution (10 mg/ml) was added, mixed by sonication and evaporated into dryness under reduced pressure at 40 °C. Then, the residue was stored in a desiccator until further evaluation. The ratio of SFN:Sylysia:HPMC:DSPE-PEG was 1:3:3:3 (w/w/w/w).

#### Preparation of physical mixture

Physical mixtures were prepared by simple intensive mixing of SFN, Sylysia, HPMC and DSPE-PEG (the ratio of SFN:Sylysia:HPMC:DSPE-PEG, 1:3:3:3, w/w/w/w) for 3 min in a mortar until a homogeneous mixture was obtained. The resulting mixtures were stored in a desiccator at room temperature until use.

### Solubility of SFN in MSNM@SFN

An excess amount of MSNM@SFN was added into 10 ml distilled water in a glass test tube. Then, the capped tube was shaken at 37 °C in a thermo statically controlled water bath for 48 h. After equilibrium had been attained, the saturated solution immediately and rapidly filtered through a 0.45 μm Millipore filter (Jingteng Science, China) and diluted with medium. Each diluted sample was analyzed by HPLC to determine the amount of dissolved SFN.

### *In vitro* release of SFN from MSNM@SFN

The release of SFN from MSNM@SFN was investigated. Briefly, MSNM@SFN (equivalent to 3 mg of SFN) was placed in 200 ml release medium (pH 1.2 containing 1% (v/v) Tween 80 or PBS pH 6.8 containing 1% (v/v) Tween 80) at 37 °C and 100 rpm. Samples (0.5 ml) were taken at predetermined time intervals from the release medium for 60 min, and replaced by a similar volume of fresh medium. Each sample was passed through a 0.45 μm millipore filter to obtain about 0.3 ml subsequent filtrate. The concentration of SFN was determined by HPLC.

### *In vivo* pharmacokinetics

Twelve male SD rats were randomly assigned to two groups (six rats per group). Group 1 received an oral administration of SFN suspension at a dose of 20 mg/kg. Group 2 received an oral administration of MSNM@SFN at a dose of 20 mg/kg SFN. After administration, approximate 0.5 ml of blood was collected by heparinized tube from the orbit venous plexus of the rat at different time points. Plasma samples were harvested by immediately centrifugation at 6000 g for 5 min and stored at −20 °C until analyzed by HPLC.

Pharmacokinetic parameters were calculated from SFN concentration-time data using noncompartmental methods as implemented by the program WinNonlin version 3.1 (Pharsight Corp., Mountain View, CA). *C*_max_ was the observed value. The AUC was calculated using the linear trapezoidal method and was extrapolated to infinity (AUC_inf_) by dividing the last measured concentration by the terminal rate constant, lz, which was determined from the slope of the terminal phase of the plasma concentration-time curve. The terminal half-life (*t*_1/2_) was calculated as 0.693 divided by lz.

### *In vitro* cytotoxicity

The cytotoxicity of MSNM@SFN against MDA-MB-231 cells was measured by sulforhodamine B assay (SRB assay). Briefly, MDA-MB-231 cells (1 × 10^4^ cells per well) were seeded in a 96-well plate and grown in medium for 24 h. Then, the cells were treated with cell culture medium containing the different amounts of MSNM@SFN for 48 h at 37 °C. The cell viability was determined by SRB assay. Absorbance was measured at 540 nm using a 96-well plate reader (model 680; Bio-Rad Laboratories, Hercules, CA). The survival percentages were calculated using the formula: survival % = (A540 nm for the treated cells/A540 nm for the control cells) × 100%, where A540 nm is the absorbance value. Each assay was carried out in triplicate. Finally, dose–effect curves were constructed and IC_50_ values were calculated.

### *In vivo* anti-tumor efficacy

The *in vivo* anti-tumor efficacy of MSNM@SFN was evaluated in MDA-MB-231 tumor-bearing nude mice (Huang et al., 2016). Briefly, female BALB/C nude mice were subcutaneously injected in the right flank with 0.2 ml cell suspension containing 1 × 10^7^ MDA-MB-231 cells with 20% basement membrane matrix. Once the tumor volume reached about 150–200 mm^3^ on the 6th day, the mice were randomly divided into three groups (7 mice per group). Then, mice were given an oral administration of physiological saline, SFN suspension or MSNM@SFN at a dose of 40 mg/kg SFN every day. The tumor volume was measured every 2 days using a calculation based on the equation (a × b^2^)/2, where a and b are the length and width of the tumor, respectively. The animals were also weighed every 2 days during the experimental period. After 32 days, all the mice were sacrificed, and the tumor tissues were removed and weighed.

Tumor sections were used for TUNEL and CD31 straining experiments. TUNEL and CD31 staining was preformed according to the standard protocols provided by the manufacturers.

### HPLC analysis of SFN

SFN in plasma samples were extracted following the method. Briefly, plasma (100 μl) was mixed with 420 μl of methanol by a vortex mixer for 30 s. The mixture was centrifuged at 3000 *g* for 10 min. A volume of 300 μl of supernatant was collected, and dried under a gentle nitrogen gas at 40 °C in a water bath. The residue was dissolved in 100 μl mobile phase as blew by vortex. The supernatant (20 μl) was injected into the HPLC system.

The HPLC system was equipped with a Waters 2487 Dual λ Absorbance Detector and 1525 pump (Waters Co., Inc., Westerville, OH). An Epic C-18 analytical column (5 μm, 250 × 4.6 mm, Phenomenex) was used and the wavelength was set at 265 nm. The mobile phase, consisting of acetonitrile–methanol–1% acetic acid (38:35:27, v/v), was delivered at a flow rate of 1 ml/min.

### Statistical analysis

All data are shown as mean ± SD unless stated otherwise. One-way analysis of variance (ANOVA) was used to determine significance among groups, after which post-hoc tests with the Bonferroni correction were used for comparisons between individual groups. Statistical significance was established at *p* < 0.05.

## Results

### Solubility of SFN in MSNM@SFN

As shown in Supplementary Table S2, the solubility of SFN in pure SFN in distilled water was under the lower detection (less than 0.1 μg/ml), however, the solubility of SFN in MSNM@SFN was 106.64 μg/ml, significantly higher than that of pure SFN.

The influence of Sylysia, HPMC, or DSPE-PEG on SFN solubility was also investigated. As shown in Supporting information and Supplementary Table S3, the solubility of SFN in the SFN-nanomatrix or SFN-HPMC solid dispersion in distilled water was under the lower detection (less than 0.1 μg/ml) or 0.9 μg/ml. The solubility of SFN in SFN-DSPE-PEG solid dispersion, SFN-Sylysia-HPMC nanomatrix or SFN-Sylysia-DSPE-PEG nanomatrix was significantly increased to 10–30 μg/ml. However, the solubility of SFN in MSNM@SFN was significantly higher than that of other SFN nanomatrixes in distilled water. In addition, this system could also significantly enhance the solubility of the poor water soluble drug paclitaxel (PTX) and 7-ethyl-10-hydroxycamptothecin (SN38), as shown in Supporting information and Supplementary Tables S4 and S5.

In addition, the characteristics of MSNM@SFN were evaluated, as shown in Supporting information and Supplementary Figures S1-S3 and Supplementary Table S1.

### Release of SFN from MSNM@SFN

**Figure 1.**
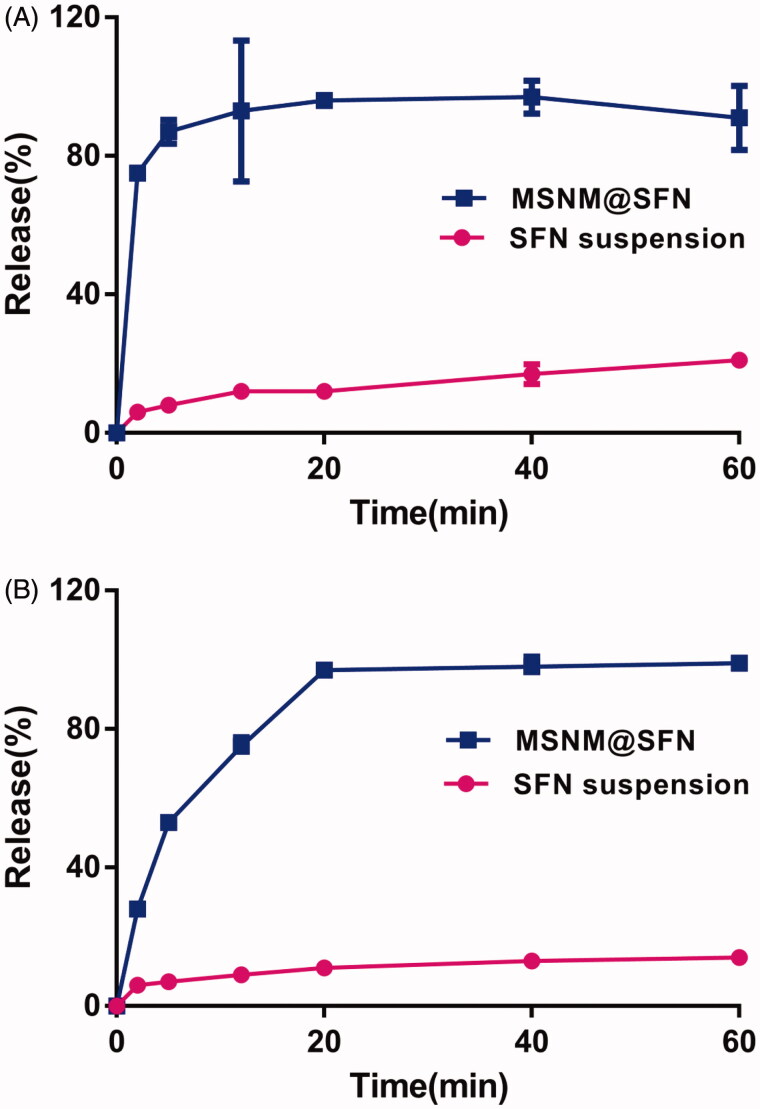
The *in vitro* release of SFN from MSNM@SFN at 37 °C in pH 1.2 (A) and 6.8 (B) PBS buffer medium. Data represent the mean ± SD (*n* = 3).

The release of SFN from MSNM@SFN is shown in Figure 1. As shown in Figure 1(A), the rapid release behavior of SFN from MSNM@SFN in pH 1.2 medium was observed. Approximately 80% of the SFN from MSNM@SFN compared to about 10% of SFN suspension was released at the time point of 5 min. In the PBS pH 6.8 medium, the released SFN from MSNM@SFN was also significantly higher than that of SFN suspension, approximately 95% of the SFN from MSNM@SFN compared to about 10% of SFN from SFN suspension was released at the time point of 20 min (Figure 1B).

### *In vivo* pharmacokinetics

**Figure 2.**
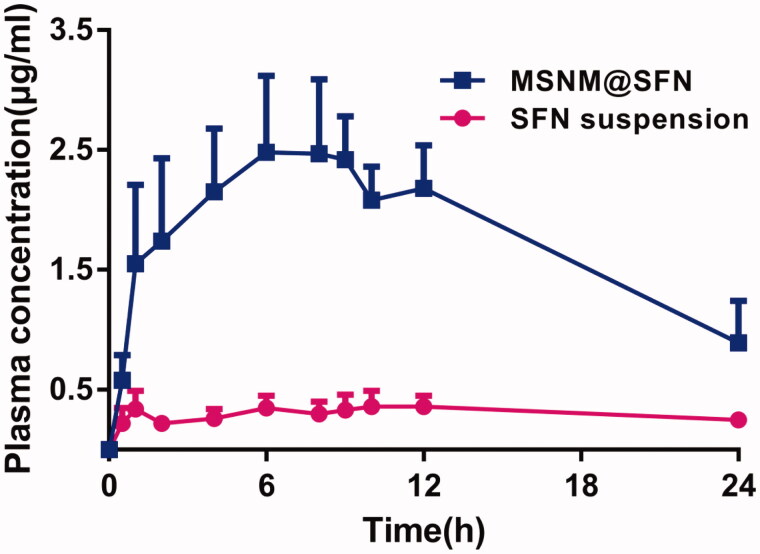
The plasma concentration-time profiles of SFN after oral administration of a single dose of SFN suspension or MSNM@SFN at 20 mg/kg SFN in SD rats (mean ± SD, *n* = 6).

The mean SFN plasma concentration-time profiles after oral administration of a single dose of SFN suspension or MSNM@SFN to rats are depicted in Figure 2. The plasma concentrations of SFN in MSNM@SFN group were significantly higher than that in SFN suspension group. The typical pharmacokinetic parameters are shown in Table 1. The results indicated that the *C*_max_ of SFN in MSNM@SFN (2.62 ± 0.56 μg/ml) was about 5.8-fold than that in SFN suspension (0.45 ± 0.08 μg/ml) (*p* < 0.01). Also, the AUC_0–24_ of SFN in MSNM@SFN (43.29 ± 10.41 h μg/ml) was about 7.7-fold than that in SFN suspension (5.61 ± 2.43 h μg/ml) (*p* < 0.01). There was no significant difference in *T*_max_, MRT_0–24 h_ and *t*_1/2_ of SFN between the SFN suspension group and the MSNM@SFN group.

**Table 1.** Pharmacokinetic parameters of SFN after oral administration of a single dose of SFN suspension or MSNM@SFN at 20 mg/kg SFN in SD rats (mean ± SD, *n* = 6).

Parameters	SFN suspension	MSNM@SFN
*C*_max_ (μg/ml)	0.45 ± 0.08	2.62 ± 0.56**
AUC_0–24_ (μg/ml·h)	5.61 ± 2.43	43.29 ± 10.41**
*T*_max_ (h)	6.40 ± 3.51	7.40 ± 3.05
MRT_0–24_ (h)	9.85 ± 3.02	10.42 ± 0.50
*t*_1/2_ (h)	9.85 ± 3.02	10.42 ± 0.50

**p < 0.01 versus the SFN suspension treatment group.

### *In vitro* cytotoxicity of MSNM@SFN

The *in vitro* cytotoxicity of MSNM@SFN was evaluated in the MDA-MB-231 cell lines. The calculated IC_50_ values are displayed in Table 2. MSNM@SFN was much more effective in inhibiting the proliferation of MDA-MB-231 compared with free SFN. The carrier material was no evident cytotoxicity on MDA-MB-231 cells (data not shown).

**Table 2.** The IC_50_ values (μM) of MSNM@SFN in MDA-MB-231 cells (mean ± SD, *n* = 3).

	Free SFN	MSNM@SFN
IC_50_ values (μM)	5.84 ± 0.17	1.45 ± 0.01**

**p < 0.01 versus the free SFN treatment group.

### *In vivo* anti-tumor activity of MSNM@SFN

**Figure 3.**
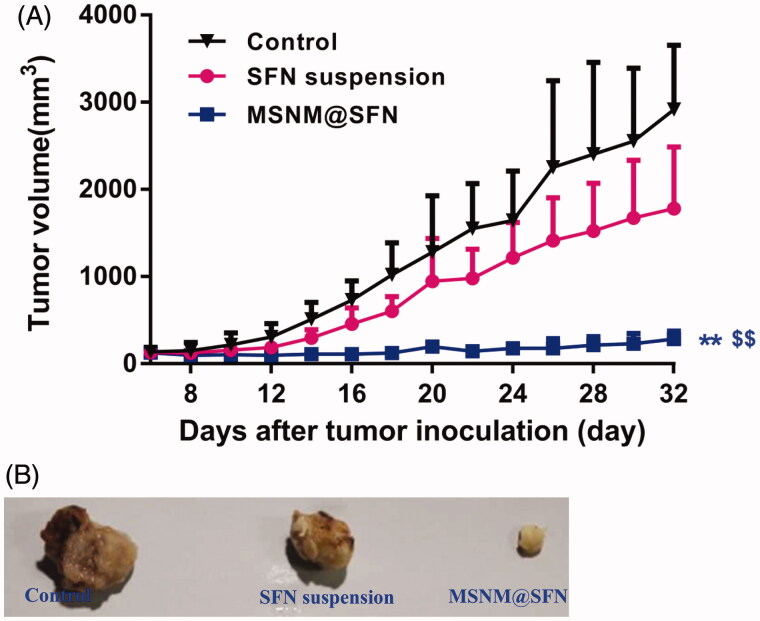
*In vivo* anti-tumor activity of MSNM@SFN in MDA-MB-231 tumor-bearing nude mice (mean ± SD, *n* = 6). BALB/C nude mice were inoculated SC with MDA-MB-231 cells and treated with physiological saline as a control, SFN suspension (40 mg/kg) and MSNM@SFN (SFN 40 mg/kg) by oral gavage every day. The tumors were measured with calipers every 2 days throughout the study. A: Tumor growth. B: The photographs of the typical tumors. **p < 0.01 versus the physiological saline treatment group as a control. ^$$^*p* < 0.01 versus the SFN suspension treatment group.

The *in vivo* anti-tumor activity of the MSNM@SFN was investigated in MDA-MB-231 tumor-bearing nude mice. As shown in Figure 3(A), the MSNM@SFN markedly inhibited the growth of MDA-MB-231 tumors (*p* < 0.01). SFN suspension has a slight anti-tumor activity which has no significant compared with control group. The anti-tumor activity of the MSNM@SFN was significantly higher than that of SFN suspension and control groups (*p* < 0.01).

The mean tumor sizes at day 32 after implantation in the SFN suspension and MSNM@SFN groups were 1783 ± 706 and 286 ± 88 mm^3^, respectively, compared with 2919 ± 735 mm^3^ in the control group. Corresponding tumor growth inhibition in the SFN suspension and MSNM@SFN groups was 38.9% and 90.2%, respectively.

The typical tumors of control, SFN suspension and MSNM@SFN group at day 32 were shown in Figure 3(B).

We also evaluated the effect of tumor cell apoptosis by TUNEL analysis staining of tumor tissue sections. As shown in Figure 4(A), tumors from the MSNM@SFN-treated group exhibited more advanced cell apoptosis compared with the groups treated with physiological saline and SFN suspension. The calculated results are shown in Figure 4(B).

Figure 4.Effects of MSNM@SFN on apoptosis and anti-angiogenesis in MDA-MB-231 tumors. A: TUNEL staining of the paraffin-embedded tumors was performed according to the standard protocols provided by the manufacturers. Tumor apoptosis cells were detected by TUNEL. DNA strand breaks were labeled (green) and nuclei were stained with Hoechst 332589 (blue). Apoptotic cells exhibited a turquoise color as a result of color merging of these two labels. B: The fluorescence area of each group was used for the statistical analysis of apoptosis activity. C: Frozen tumor sections were examined by confocal microscopy. Blood vessels were labeled with anti-CD31 (green), and nuclei were stained with DAPI (blue). D: The fluorescence area of each group was used for the statistical analysis of apoptosis activity. **p < 0.01 versus the physiological saline treatment group as a control. ^$$^*p* < 0.01 versus the SFN suspension treatment group.

**Figure:**
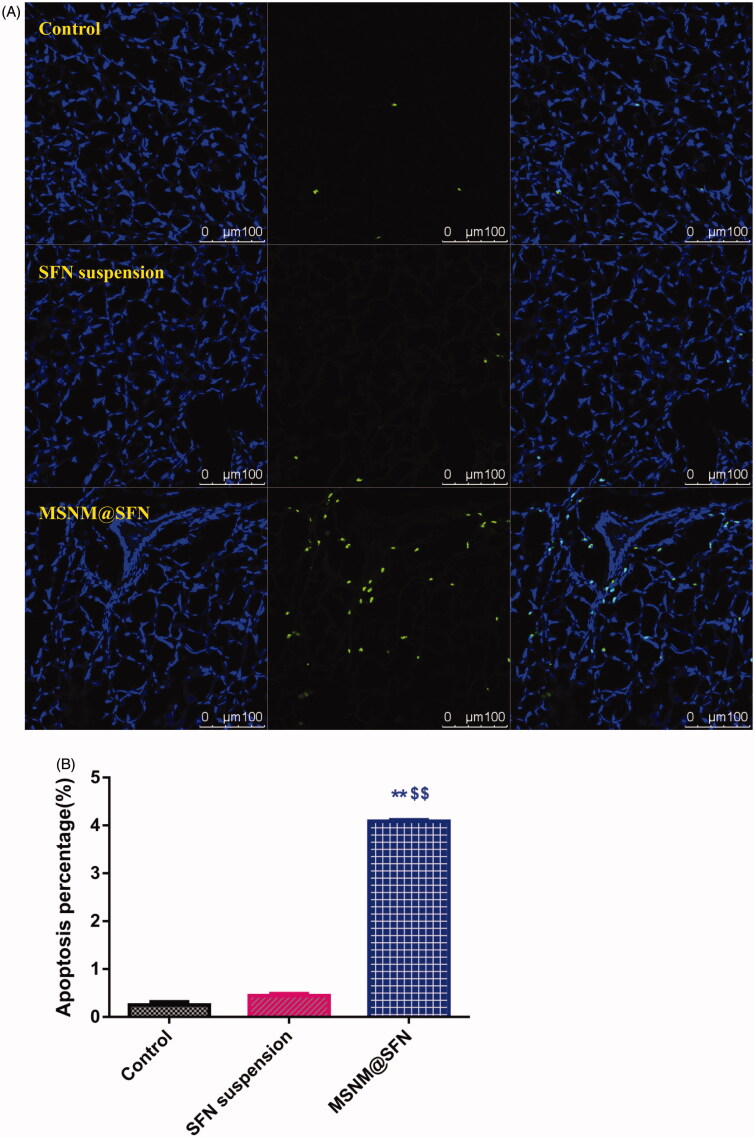

**Figure:**
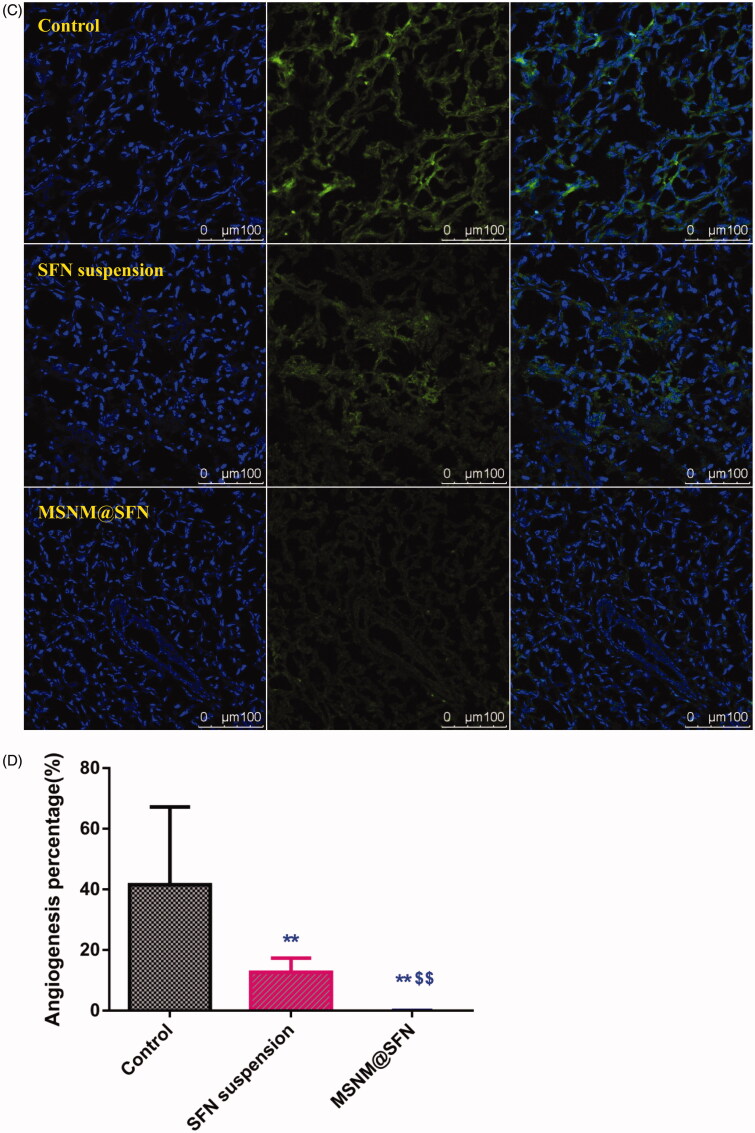

To evaluate the anti-angiogenic activity of MSNM@SFN treatment *in vivo*, the microvessel density was assessed by immunohistochemistry. As shown in Figure 4(C), microvessels were clearly observed by CD31 staining. Few microvessels were observed in SFN suspension treatment group. The microvessels in the MSNM@SFN treatment group were significantly less than those in SFN suspension or control groups. The calculated results are shown in Figure 4(D).

## Discussion

SFN is slightly absorbed in the GI due to its poor solubility in water. It has been reported that mesoporous materials, such as, mesoporous silica or mesoporous carbon, could be used as poorly water-soluble drug carriers (Jia et al., 2011; Minagawa et al., 2012; Dai et al., 2015; Zhang et al., 2015). Usually, nanomatrix were prepared by absorbed the poorly water-soluble drug on the surface or in the nano-structure of the mesoporous material and then coated with suitable excipients. This nanomatrix could significantly increase the poorly water-soluble drug solubility and oral bioavailability. In the present research, we used Sylysia 350 as mesoporous material, DSPE-PEG as absorption enhancer and HPMC as crystallization inhibitor to prepare SFN nanomatrix (MSNM@SFN). HPMC is a highly water-soluble polymer which has been found to be effective crystallization inhibitor for amorphous forms in solid states during storage or in liquid states during dissolution process (Shi et al., 2014). It has been reported that lipids can enhance drug solubilization in the intestinal milieu, interact with enterocyte-based transport or alter the pathway of drug transport to the systemic circulation to enhance the poorly water-soluble drug absorption and bioavailability (Porter et al., 2007). Interestingly, our results indicated that the combined utilization of HPMC and DSPE-PEG, as a suitable ratio, could significantly increase the solubility of SFN than that of HPMC or DSPE-PEG single used as coated material, showing the synergistic effect of HPMC and DSPE-PEG on enhancing SFN solubility.

Due to hydrophobicity, SFN has a strong tendency to crystallize which could be considered as a major obstacle for its dissolving and dissolution (Wang et al., 2011). Our DSC and PXRD results indicated that the state of SFN in the MSNM@SFN is an amorphous form which could lead to enhance its solubility and dissolution (Alonzo et al., 2010; Raina et al., 2014). The crystallization inhibition effect of HPMC could also result in the greatest degree of maintaining a supersaturated state that can sustain most effectively for biologically relevant timeframes.

For oral administration, the therapeutic efficacy of drugs significantly depends on their oral bioavailability (Stuurman et al., 2013). Our *in vivo* bioavailability results indicated the AUC of SFN in MSNM@SFN was about 7.7-fold than that in SFN suspension, indicating that the greatly improved oral bioavailability of SFN could mainly ascribe to the markedly increased solubility and dissolution, and obviously enhanced absorption effect of DSPE-PEG. Thereby, the greatly enhanced oral bioavailability of SFN in MSNM@SFN could be promising for improving the therapeutic efficacy. Our *in vivo* anti-tumor activity results indicated that MSNM@SFN markedly inhibited the growth of MDA-MB-231 tumors compared with SFN suspension. Meanwhile, the anti-microvessels effect produced by MSNM@SFN was also significantly higher than that produced by SFN suspension.

In addition, since the Sylysia 350 was not able to be absorbed into the systemic circulation, we suggested that the MSNM@SFN could have a favorable biosafety for further preclinical translation. Moreover, this nanomatrix system could also significantly enhance the solubility of the poorly water-soluble drug paclitaxel (PTX) and 7-ethyl-10-hydroxycamptothecin (SN38), indicating that this nanomatrix could be a promising drug delivery platform for improving the oral bioavailability and therapeutic efficacy of poorly water-soluble drugs.

## Conclusion

In the present study, we select Sylysia 350 as mesoporous material, DSPE-PEG as an absorption enhancer and HPMC as a crystallization inhibitor to prepare MSNM@SFN for improving the anti-tumor activity of SFN. Our results indicated that the SFN, which dispersed within the Sylysia pore or absorbed on the Sylysia surface, in MSNM@SFN was amorphous form. The solubility, dissolution, and oral bioavailability of SFN in MSNM@SFN were significantly increased. The enhanced anti-tumor activity of MSNM@SFN was confirmed *in vitro* and *in vivo* experiments. This nanomatrix developed in this study could be a promising drug delivery platform for improving the oral bioavailability and therapeutic efficacy of poorly water-soluble drugs.

## Supplementary Material

Supporting_information_-_2016-08-29.docx

## Notes

**Supplementary material available online**
