# Supplementary material for: Improving anti-tumor activity of sorafenib tosylate by lipid- and polymer-coated nanomatrix
Source: Drug Deliv. 2017 Feb 6;24(1):270–7. doi: 10.1080/10717544.2016.1245371 (PMC8241045; doi:10.1080/10717544.2016.1245371)
Supplement: Supporting_information_-_2016-08-29.docx [file IDRD_A_1245371_SM4177.docx]

**Supporting information**

**Methods**

**Characterization of MSNM@SFN**

**Scanning electron microscope (SEM)** SEM analysis was carried out using a Jeol JSM-5600LV scanning electron microscope (NOVA NANOSEM 430, FEI, USA). Prior to examination, samples were gold sputter-coated to render them electrically conductive.

**Powder X-ray diffraction (PXRD)** The powder X-ray diffraction patterns were obtained with a Rigaku Dmax/2400 apparatus (D/MAX-2000 X, Rigaku Co. Japan) using Cu-K_α_ radiation (λ=1.541 nm), a voltage of 40 kV and a 100 mA current. Samples were scanned from 3-40° 2θ for qualitative studies and the scanning rate was 4°/min.

**Differential scanning calorimetry (DSC)** The DSC studies were conducted using a Thermal Analysis DSC-Q100 differential scanning calorimeter (Thermal Analysis Co., USA). Samples of about 5 mg (± 0.5 mg) were encapsulated in flat-bottomed aluminum pans. The thermograms were recorded at a heating rate of 10 °C/min from 10 to 220 °C using nitrogen as the purging gas.

**Brunauer-Emmett-Teller (BET) and Barrett-Joyner-Halenda (BJH) study** The specific surface area, the pore size and the pore volume were determined according to the BET and the BJH method using an ASAP2010 rapid surface area and pore size analyzer (Micromeritics Co., USA). All samples were degassed at 70 °C under vacuum for 24 h prior to analysis.

**Preparation of SFN-HPMC solid dispersion**

The SFN-HPMC solid dispersion was prepared by solvent evaporation method. Briefly, a volume of 1 ml SFN methanol solution (10 mg/ml) was mixed with 10 ml HPMC dichloromethane solution (3 mg/ml) by sonication and evaporated into dryness under reduced pressure at 40°C. Then, the residue was stored in a desiccator until further evaluation. The ratio of SFN:HPMC was 1:3 (w/w).

**Preparation of SFN-DSPE-PEG solid dispersion**

The SFN-DSPE-PEG solid dispersion was prepared by solvent evaporation method. Briefly, a volume of 1 ml SFN methanol solution (10 mg/ml) was mixed with 3.0 ml of DSPE-PEG dichloromethane solution (10 mg/ml) by sonication and evaporated into dryness under reduced pressure at 40°C. Then, the residue was stored in a desiccator until further evaluation. The ratio of SFN: DSPE-PEG was 1:3 (w/w).

**Preparation of SFN-nanomatrix**

The SFN-nanomatrix was prepared by solvent evaporation method. Briefly, a volume of 1 ml SFN methanol solution (10 mg/ml) was dropped into Sylysia 350 dichloromethane solution (3 ml, 10 mg/ml) and then mixed in a round flask by sonication for 30 min. After that, the solvent was evaporated into dryness under reduced pressure at 40°C. Then, the residue was stored in a desiccator until further evaluation. The ratio of SFN:Sylysia was 1:3 (w/w).

**Preparation of SFN-HPMC nanomatrix**

The SFN-HPMC nanomatrix was prepared by solvent evaporation method. Briefly, a volume of 1 ml SFN methanol solution (10 mg/ml) was dropped into Sylysia 350 dichloromethane solution (3 ml, 10 mg/ml) and then mixed in a round flask by sonication for 30 min. After that, a volume of 10 ml HPMC dichloromethane solution (3 mg/ml) was dropped into the mixtures and stirred for 24 h and then evaporated into dryness under reduced pressure at 40°C. Then, the residue was stored in a desiccator until further evaluation. The ratio of SFN:Sylysia:HPMC was 1:3:3 (w/w/w).

**Preparation of SFN-DSPE-PEG nanomatrix**

The SFN-DSPE-PEG nanomatrix was prepared by solvent evaporation method. Briefly, a volume of 1 ml SFN methanol solution (10 mg/ml) was dropped into Sylysia 350 dichloromethane solution (3 ml, 10 mg/ml) and then mixed in a round flask by sonication for 30 min. After that, a volume of 3.0 ml of DSPE-PEG dichloromethane solution (10 mg/ml) was added, mixed by sonication and evaporated into dryness under reduced pressure at 40°C. Then, the residue was stored in a desiccator until further evaluation. The ratio of SFN:Sylysia:DSPE-PEG was 1:3:3 (w/w/w).

**Preparation of MSNM@PTX**

The MSNM@PTX was prepared by solvent evaporation method. Briefly, a volume of 1 ml paclitaxel (PTX) methanol solution (10 mg/ml) was dropped into Sylysia 350 dichloromethane solution (3 ml, 10 mg/ml) and then mixed in a round flask by sonication for 30 min. After that, a volume of 10 ml HPMC dichloromethane solution (3 mg/ml) was dropped into the mixtures and stirred for 24 h and then evaporated into dryness under reduced pressure at 40°C. Subsequently, a volume of 3.0 ml of DSPE-PEG dichloromethane solution (10 mg/ml) was added, mixed by sonication and evaporated into dryness under reduced pressure at 40°C. Then, the residue was stored in a desiccator until further evaluation. The ratio of PTX:Sylysia:HPMC:DSPE-PEG was 1:3:3:3 (w/w/w/w).

**Preparation of MSNM@SN38**

The MSNM@SN38 was prepared by solvent evaporation method. Briefly, a volume of 1 ml 7-Ethyl-10-hydroxycamptothecin (SN38) methanol solution (10 mg/ml) was dropped into Sylysia 350 dichloromethane solution (3 ml, 10 mg/ml) and then mixed in a round flask by sonication for 30 min. After that, a volume of 10 ml HPMC dichloromethane solution (3 mg/ml) was dropped into the mixtures and stirred for 24 h and then evaporated into dryness under reduced pressure at 40°C. Subsequently, a volume of 3.0 ml of DSPE-PEG dichloromethane solution (10 mg/ml) was added, mixed by sonication and evaporated into dryness under reduced pressure at 40°C. Then, the residue was stored in a desiccator until further evaluation. The ratio of SN38:Sylysia:HPMC:DSPE-PEG was 1:3:3:3 (w/w/w/w).

**Results**

**Characterization of MSNM@SFN**

**Scanning electron microscope (SEM)** The SEM images of the pure SFN, Sylysia and MSNM@SFN were shown in Fig. S1. Pure SFN was observed as needle or rod-like crystals that formed aggregates ranges from 100-500 μm. Sylysia was seen as sphere shape of particles (about 3 μm). However, no SFN crystals were observed in MSNM@SFN, indicating that SFN was dispersed within the Sylysia pore or absorbed on the Sylysia surface.

**Differential scanning calorimetry (DSC)** The DSC thermograms of pure SFN, Sylysia, HPMC, DSPE-PEG, physical mixture and MSNM@SFN are shown in Fig. S2A. There was no endothermic peak observed in HPMC or Sylysia. The pure SFN curve showed a wide endothermic peak at about 196.75°C. For pure DSPE-PEG, a sharp endothermic peak was at about 55.95°C. The endotherm peaks of SFN and DSPE-PEG were still observed in physical mixture. The complete disappearance of SFN or DSPE-PEG endothermic peaks was observed in MSNM@SFN.

**Powder X ray diffraction (PXRD)** The PXRD patterns for pure SFN, Sylysia, HPMC, DSPE-PEG, physical mixture and MSNM@SFN were shown in Fig. S2B. In the X-ray diffraction spectrum of pure SFN, some sharp and intense peaks at a diffraction angle of 2θ 4.36°, 13.20°, 24.60° were observed, showing that SFN was present as a crystalline material. The peaks of DSPE-PEG at 2θ values were also observed at 18.92° and 23.16°. There was no evident peak observed in Sylysia or HPMC. For physical mixture, some SFN or DSPE-PEG crystallinity peaks were also detectable. In contrast, there was no sharp peak attributable to SFN or DSPE-PEG in the MSNM@SFN, suggesting that SFN in this MSNM@SFN was in amorphous stat.

**Brunauer-Emmett-Teller (BET) and Barrett-Joyner-Halenda (BJH) analysis** BET and BJH were used to calculate the specific surface area, the pore volume and size, respectively. As shown in Fig. S3, the pore size distribution of MSNM@SFN was significant lower than that of Sylysia. Also, the BJH surface area, pore volume and pore diameter of MSNM@SFN were significant lower than those of Sylysia, as shown in Table S1, indicated that the some of the SFN might enter into the nanopores of Sylysia.

**Solubility of SFN**

The solubility of SFN in the SFN-nanomatrix or SFN-HPMC solid dispersion in distilled water was under the lower detection (less than 0.1 µg/ml). The solubility of SFN in SFN-DSPE-PEG solid dispersion, SFN-Sylysia-HPMC nanomatrix or SFN-Sylysia-DSPE-PEG nanomatrix was significant increased to 10-30 μg/ml. However, the solubility of SFN in MSNM@SFN was significant higher than that of other SFN nanomatrixes in distilled water, as shown in Table S3. In addition, this system could also significant enhance the solubility of the poor water soluble drug paclitaxel (PTX) and 7-Ethyl-10-hydroxycamptothecin (SN38), as shown in Table S4 and S5.


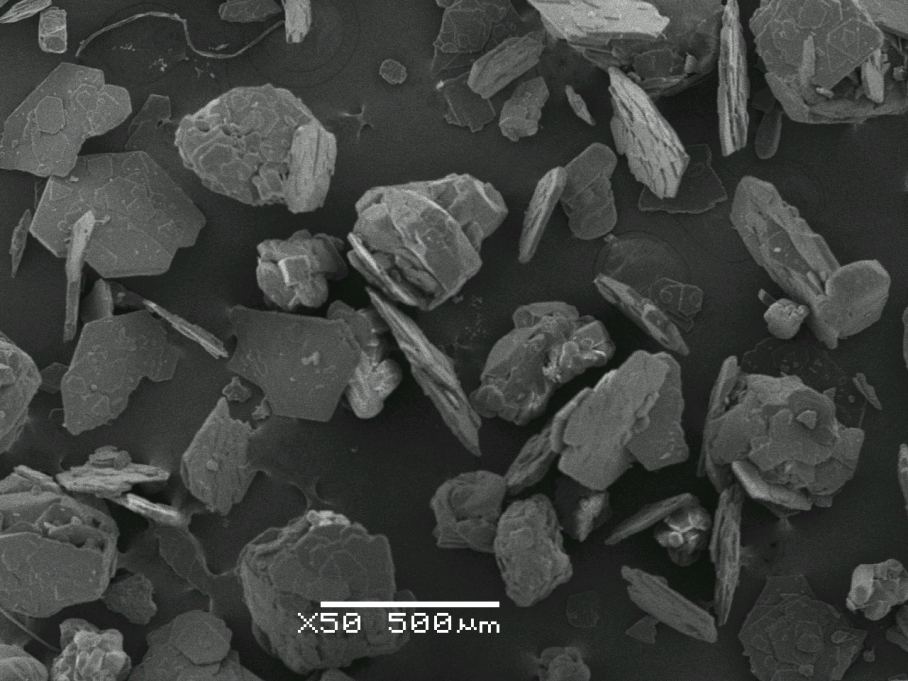


**SFN**


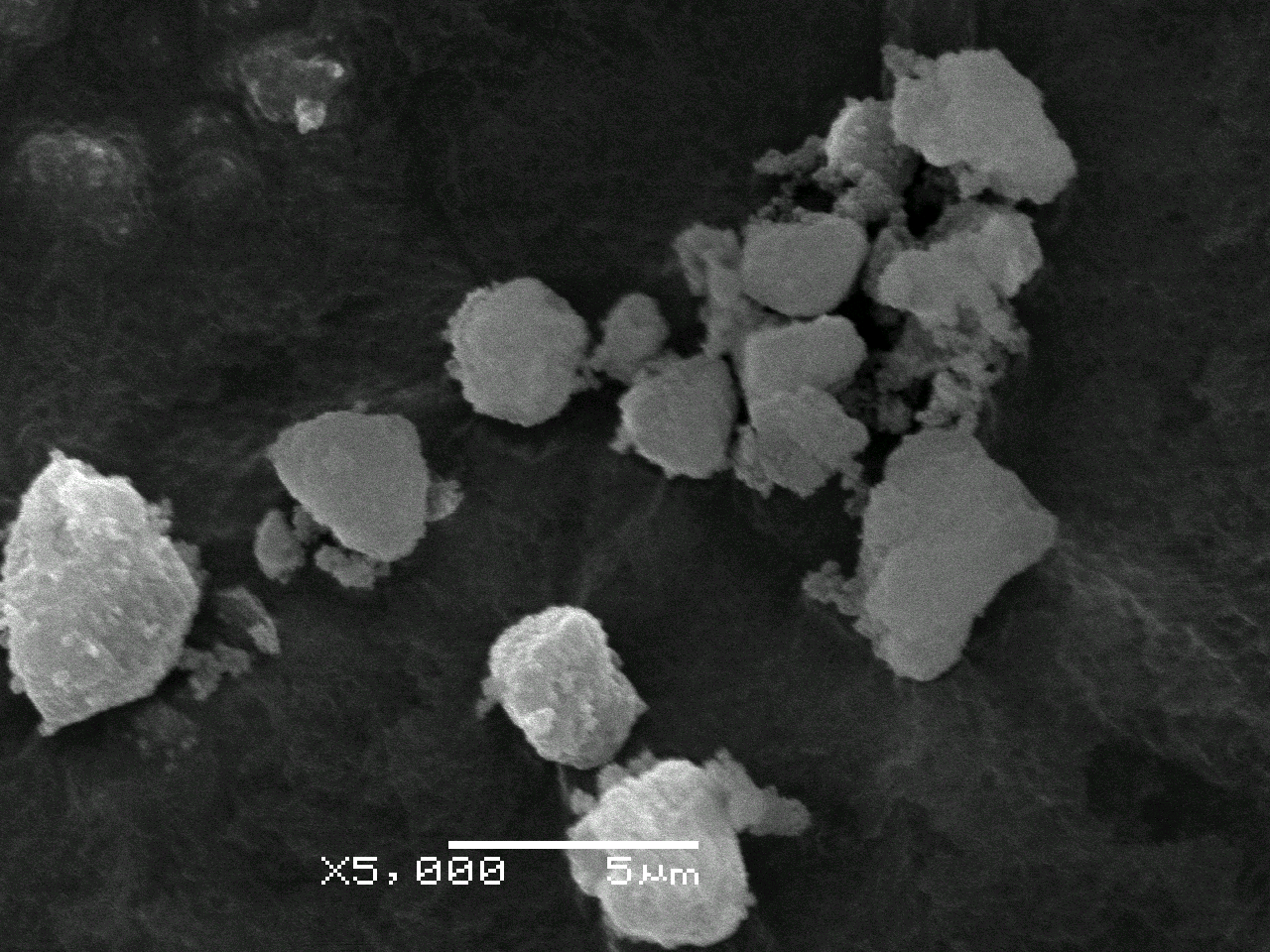


**Sylysia**


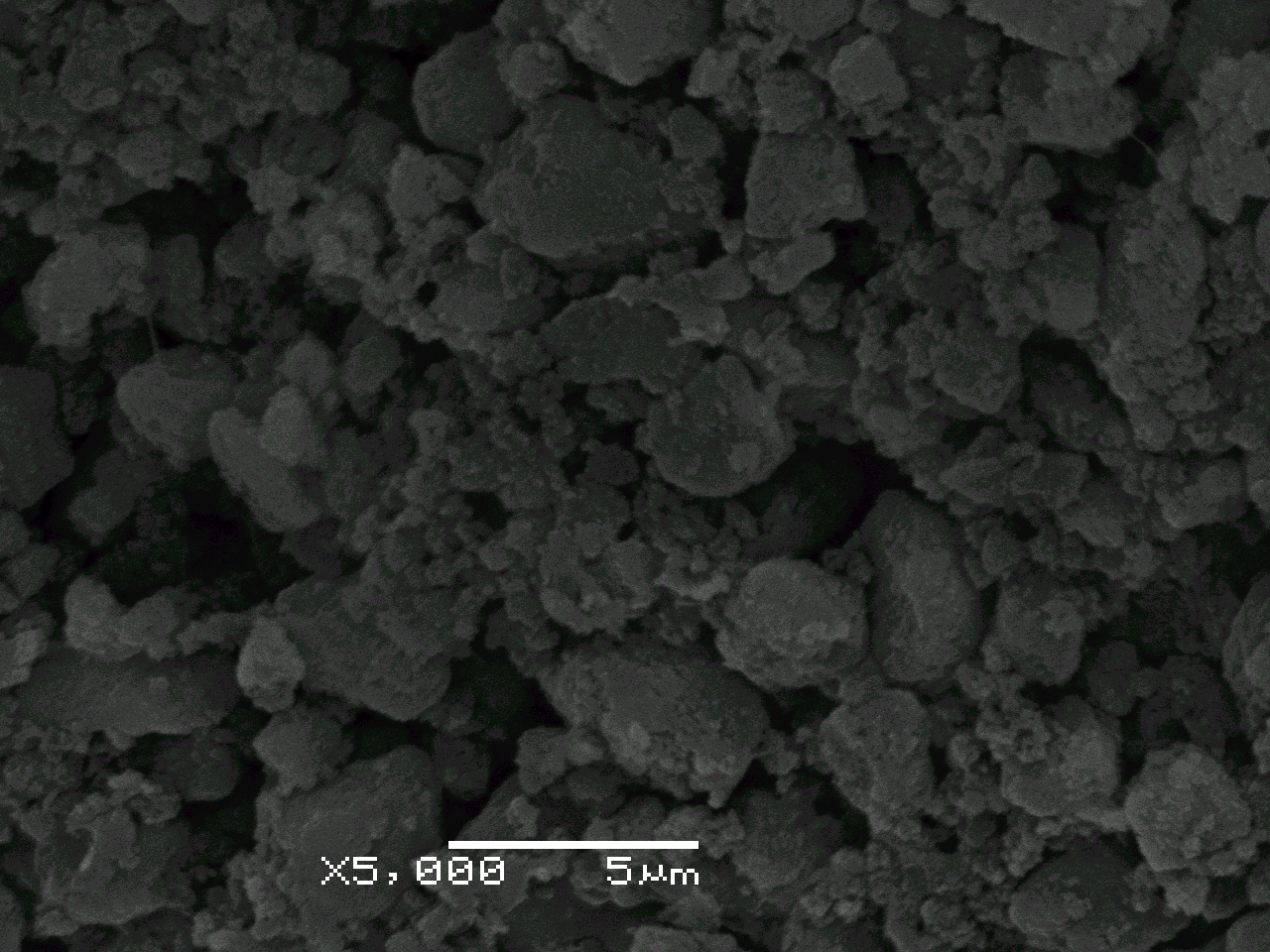


**MSNM@SFN**

Fig.S1. Topical SEM images of pure SFN, Sylysia and MSNM@SFN.


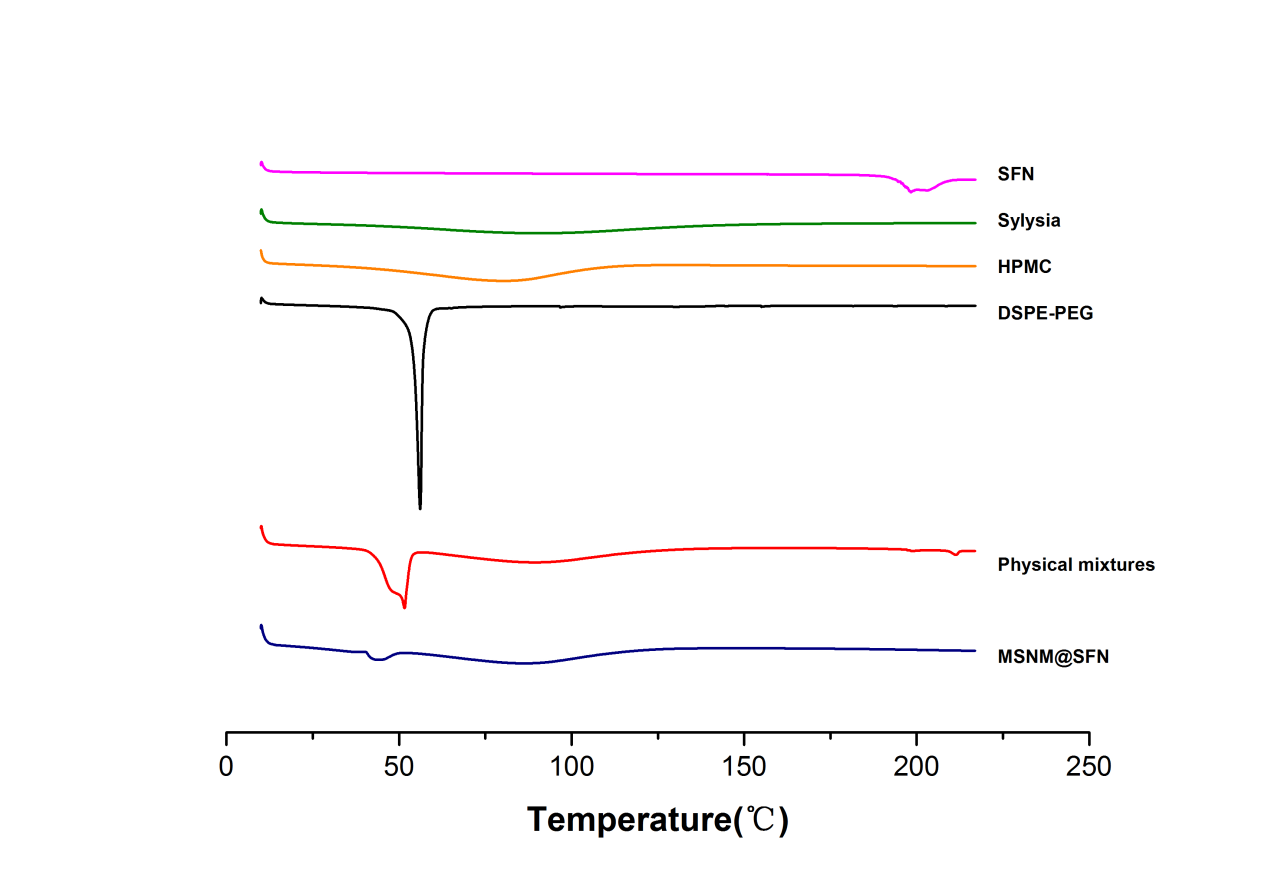


**A**

**
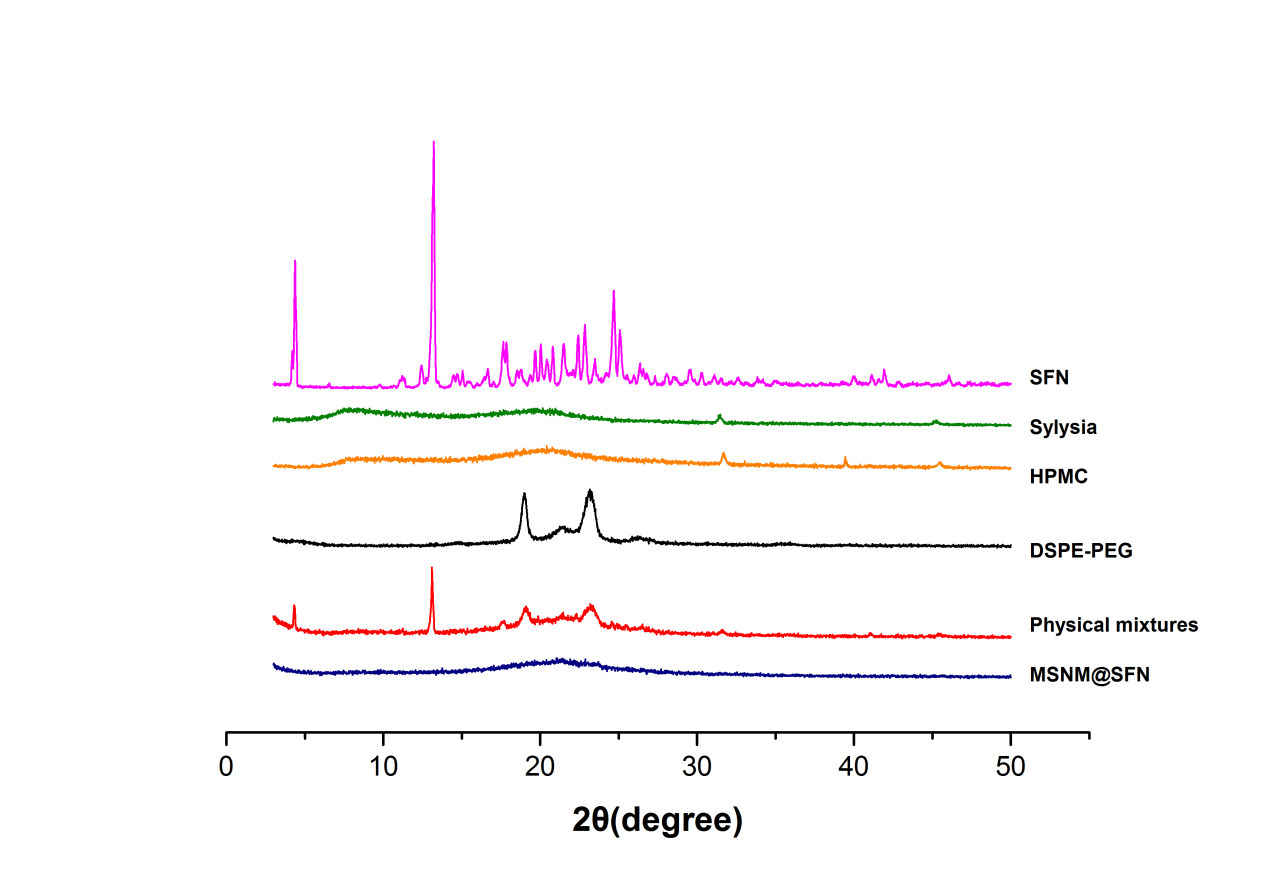
**

**B**

Fig.S2. The DSC thermograms (A) and PXRD patterns (B) of pure SFN, Sylysia, HPMC, DSPE-PEG, physical mixture and MSNM@SFN.

Fig.S3. BJH pore size distribution curves of Sylysia and MSNM@SFN.

Table S1. Specific surface area, pore volume and pore diameter of Sylysia and MSNM@SFN.

|  | Surface Area (cm³/g) | Pore Volume (cm³/g) | Pore Size (nm) |
| --- | --- | --- | --- |
| Sylysia | 344.16 | 1.65 | 19.22 |
| MSNM@SFN | 42.84 | 0.17 | 15.43 |

Table S2. The solubility of SFN in MSNM@SFN.

|  | Solubility (μg/ml) |
| --- | --- |
| SFN | <0.1 |
| MSNM@SFN (SFN:Sylysia:HPMC:DSPE-PEG=1:3:3:3) | 106.64±16.60 |

Table S3. The solubility of SFN.

|  | Solubility(μg/ml) |
| --- | --- |
| SFN | ＜0.1 |
| SFN-nanomatrix (SFN:Sylysia=1:3, w/w) | ＜0.1 |
| SFN-HPMC solid dispersion (SFN:HPMC=1:3, w/w) | 0.93±0.02 |
| SFN-DSPE-PEG solid dispersion (SFN:DSPE-PEG=1:3, w/w) | 13.51±0.03 |
| SFN-HPMC nanomatrix (SFN: Sylysia:HPMC=1:3:3, w/w/w) | 11.83±1.69 |
| SFN-DSPE-PEG nanomatrix (SFN: Sylysia:DSPE-PEG=1:3:3, w/w/w) | 27.49±3.84 |
| MSNM@SFN (SFN: Sylysia:HPMC:DSPE-PEG=1:3:3:3, w/w/w/w) | 106.64±16.60 |

Table S4. The solubility of PTX.

|  | Solubility(μg/ml) |
| --- | --- |
| PTX | 0.17±0.02 |
| MSNM@PTX (PTX: Sylysia:HPMC:DSPE-PEG=1:3:3:3) | 129.24±57.27 |

Table S5. The solubility of SN38.

|  | Solubility(μg/ml) |
| --- | --- |
| SN38 | ＜0.01 |
| MSNM@SN38 (SN38: Sylysia:HPMC:DSPE-PEG=1:3:3:3, w/w/w/w) | 9.41±0.97 |
